# Supplementary material for: A Structural Proteomics Exploration of Synphilin-1 and Alpha-Synuclein Interaction in Pathogenesis of Parkinson’s Disease
Source: Biomolecules. 2024 Dec 12;14(12):1588. doi: 10.3390/biom14121588 (PMC11674031; doi:10.3390/biom14121588)
Supplement: Supplementary file 1 [file biomolecules-14-01588-s001.zip › biomolecules-3304576-supplementary.pdf]

Supplementary Table S1. A comparative profile of binding profiles of interactions obtained for individual proteins using original structure, DTS and DAA models respectively. In the interaction details, the residues (in single letter) are given in bracket after domain indices.

Proteins (PDB ID)	Ligands (Chemical ID)	Total number of domains	Interactions details<br>Residues (single letter with chain index) in bracket after the domain indices		
			**Experimental**	**DAA**	**DTS**
Mutant monomer of recombinant human hexokinase Type I complexed with Glucose, Glucose-6-Phosphate, and ADP(1CZA)	alpha-D-glucose 6-phosphate<br>(G6P)	8	**2** (S88,G87,D84,D209);<br>**3** (T232,G231)<br>**4**(G448,S449)	**2**(S88,G86,G87,R91, K173);<br>**3**(T232,G233);<br>**4**(S449)	**2**(S88,G87,D84, ,D209. K173);<br>**3**(T232);<br>**4**(S449)
Crystal structure of the aldehyde oxidoreductase from desulfovibriodesulfuricansatcc 27774 (1DGJ)	Pterin cytosine dinucleotide<br>(MCN)	2	**1**(F423, T422);<br>**2**(V804, N800, C799, K797,G662,Q703,G658,S700,A697,S702, W652,Q657,)	**2**(S821,K829,G834,G835)	**2**(S840,T832,K842)
Crystal structure of human leucyl-tRNA synthetase, Leu-AMS-bound form(6KIE)	5'-O-(L-leucylsulfamoyl)adenosine<br>(LSS)	4	**1**(H103, Y64, Y66, H75, H72, S78);<br>**2**(H263, H693, S685, S686, D688, L722, G720)	**2**(N572, E568, K687, P691)	**2**(K687, K728)

Structure determinants of phosphoinositide 3-kinase inhibition by wortmannin, LY294002, quercetin, myricetin and staurosporine (1E7U)	Wortmannin (KWT)	5	**4**(M663,P669,I690,S665,Y726); **5**(D809,D823)	**5**(G829,N838,E840)	**5**(D912, N930,Y926)
X-ray crystal structure of human ceruloplasmin at 3.0 angstroms (1KCW)	2-acetamido-2-deoxy-beta-D-glucopyranose (NAG)	4	**2**(K288); **4**(Y986, D998)	**2**(N296); **4**(D998)	**2**(I295, N296); **4**(D998)
Crystal structure of endoplasmic reticulum aminopeptidase 2 (erap2) complex with a highly selective and potent small molecule (7SH0)	(2S)-N-hydroxy-3-(4-methoxyphenyl)-2-[4-({[5-(pyridin-2-yl)thiophene-2-sulfonyl]amino}methyl)-1H-1,2,3-triazol-1-yl]propanamide (GIY)	3	**1**(E200); **2**(Y455, P333, A335, H370, W363, E400); **3**(Y892)	**1**(E177, E200); **2**(A332, A335, G334, Y455, P333)	**2**(K397, E423, P333, Y455, E400, F450, H370)
Omecamtiv Mercarbil binding site on the Human Beta-Cardiac Myosin Motor Domain (4PA0)	methyl 4-(2-fluoro-3-{[(6-methylpyridin-3-yl)carbamoyl]amino}benzyl)piperazine-1-carboxylate (2OW)	3	**2**(A91, M92, S118, C705, P710, N711, R712, L770)	**2**(I87, E88, D89, R712, L714, K762)	**2**(L120, F121, C122, I174, L175, I157, V670)

Structure of human DNMT1 (601-1600) in complex with Sinefungin(3SWR)	SINEFUNGIN (SFG)	5	**3**(L649, P627, E668)<br>**4**(V982)	**1**(E113)<br>**4**(L759)	**4**(I756, L759)
Structure of the m1 alanylaminopeptidase from malaria complexed with a hydroxamic acid-based inhibitor(4R5X)	3-amino-N-{(1R)-2-(hydroxyamino)-2-oxo-1-[4-(1H-pyrazol-1-yl)phenyl]ethyl}benzamide(R5X)	4	**1**(H315,H319,E338)	**2**(N459,L463,Q460,T532)	**2**(S455,N459,L463,K504, P505)
Structure of Ca2+ ATPase(5ZTF)	Phosphomethylphosphonic acid adenylate ester (ACP)	3	**2**(K380,T381,R517,E470,M522,K542,G543,R587,G653,K711,R705,N733,F514)	**2**(T585,L586,R587,N655)	**2**(A468,T469,R587,K464,D628,D654,T585)
Structure of C-terminal fragment of Vip3A toxin(6VLS)	DI(HYDROXYETHYL)ETHER (PEG)	5	**2**(W250)<br>**3**(L396,E398)	**1**(D15,K16,E112)<br>**2**(P299,L300)	**1**(D15, K16, E112, N13)
E.coli beta-galactosidase (E537Q) in complex with fluorescent probe KSA02(7BRS)	8-[2-[(E)-2-[4-[(2S,3R,4S,5R,6R)-6-(hydroxymethyl)-3,4,5-tris(oxidanyl)oxan-2-yl]oxyphenyl]ethenyl]-3,3-dimethyl-indol-1-ium-1-yl]octanoic acid (F4X)	5	**1**(N104, V105, D203) **3**(E463, Q539, H542, M504, N606)<br>**5**(V797,W1001)	**1**(N104)<br>**3**(E463, M504, V517, V423, P424,M425, F514)<br>**5**(S798,R802)	**1**(N104,V105, D203)<br>**3**(E463, Q539, H542, Y505, F514, A516, H393)<br>**5**(V797, W1001, S798)

Crystal structure of human MTR4 (6IEG)	ADENOSINE-5'-DIPHOSPHATE (ADP)	5	**1**(S164, F138, Q144, V169, I139, T168, G166, K167)	**1**(D219); **2**(E476, K463, E443, T442 G417); **5**(R955)	**1**(L175, I174, I157) **4**(V670)
Structure of human sodium-calciumexchanger NCX1(8JP0)	2-{4-[(2,5-difluorophenyl)methoxy]phenoxy}-5-ethoxyaniline (EKY)	5	**1**(V134, H200, F248, W210 C245) **5**(D828, A829, A839, G836)	**1**(E110, T126); **2**(A322, V353, Q357); **4**(I694, N698)	**2**(K340, P338, I342, K334, Q335)

Supplementary Table S2. The residue count for each of the fragments of all the proteins (both training and syn-1

Proteins	Sequence length	The residue count for each of the fragments		
		Fragment1	Fragment2	Fragment3
1CZA	917	1 to 400	301 to 700	601 to 917
1DGJ	907	1 to 400	301 to 700	601 to 907
6KIE	1073	1 to 400	301 to 700	601 to 1073
1E7U	961	1 to 400	301 to 700	601 to 961
1KCW	1046	1 to 400	301 to 700	601 to 1046
7SH0	967	1 to 400	301 to 700	601 to 967
4PA0	1024	1 to 400	301 to 700	601 to 1024
3SWR	1002	1 to 400	301 to 700	601 to 1002
4R5X	903	1 to 400	301 to 700	601 to 903
5ZTF	1070	1 to 400	301 to 700	601 to 1070
6VLS	966	1 to 400	301 to 700	601 to 966
7BRS	1025	1 to 400	301 to 700	601 to 1025
6IEG	1002	1 to 400	301 to 700	601 to 1002
8JP0	937	1 to 400	301 to 700	601 to 937
Syn-1	919	1 to 400	301 to 700	601 to 919

Supplementary MATLAB Code: For impletentation of fragmentation of part of divide and conquer algorithm (comments are made using '%' sign)

```
function [pr_frgmts, res_range1] = do_frgmt(pr_seq)

%The function do_frgmt implements the fragmentation of protein sequence,
%pr_seq following the fragmentation algorithm as given below. The output,
%pr_frgmts is the cell variable containing all the fragments and res_range1
is %the residue range for each of the fragments.

%Computation of number of fragments, frg_no considering all the fragment size
%as 400 except the last fragment that is compensated depending on the size of
%the protein sequence, pr_seq.

frg_no = ceil((length(pr_seq)/400) + 0.01);
n = frg_no;
l = length(pr_seq);

res_range1 =[];
for i = 1:n
    if i==1
frgi = pr_seq(400*(i-1)+1:400*i);
res_range = [400*(i-1)+1 400*i];

    elseif (i<n)+(i>1) == 2
frgi = pr_seq(400*(i-1)-100*(i-1)+1:400*i-100*(i-1));
res_range = [400*(i-1)-100*(i-1)+1 400*i-100*(i-1)];
    elseif i==n
frgi = pr_seq(400*(i-1)-100*(i-1)+1:l);
res_range = [400*(i-1)-100*(i-1)+1 l];
    end
pr_frgmts{i} = frgi;
    res_range1 = [res_range1;res_range];
end

end
```
